# A Scoping Review of Attitudes and Experiences with Pharmacogenomic Testing among Patients and the General Public: Implications for Patient Counseling

**DOI:** 10.3390/jpm12030425

**Published:** 2022-03-09

**Authors:** Josiah D. Allen, Amy L. Pittenger, Jeffrey R. Bishop

**Affiliations:** 1 Department of Experimental and Clinical Pharmacology, College of Pharmacy, University of Minnesota, Minneapolis, MN 55455, USA; alle0861@umn.edu; 2 Medigenics Consulting, LLC, Minneapolis, MN 55407, USA; 3 Department of Pharmaceutical Care and Health Systems, College of Pharmacy, University of Minnesota, Minneapolis, MN 55455, USA; alp@umn.edu; 4 Department of Psychiatry and Behavioral Sciences, Medical School, University of Minnesota, Minneapolis, MN 55455, USA

**Keywords:** pharmacogenomics, patient experience, patient counseling

## Abstract

The use of pharmacogenomic (PGx) tests is increasing, but there are not standard approaches to counseling patients on their implications or results. To inform approaches for patient counseling, we conducted a scoping review of published literature on patient experiences with PGx testing and performed a thematic analysis of qualitative and quantitative reports. A structured scoping review was conducted using Joanna Briggs Institute guidance. The search identified 37 articles (involving *n* = 6252 participants) published between 2010 and 2021 from a diverse range of populations and using a variety of study methodologies. Thematic analysis identified five themes (reasons for testing/perceived benefit, understanding of results, psychological response, impact of testing on patient/provider relationship, concerns about testing/perceived harm) and 22 subthemes. These results provide valuable context and potential areas of focus during patient counseling on PGx. Many of the knowledge gaps, misunderstandings, and concerns that participants identified could be mitigated by pre- and post-test counseling. More research is needed on patients’ PGx literacy needs, along with the development of a standardized, open-source patient education curriculum and the development of validated PGx literacy assessment tools.

## 1. Introduction

Pharmacogenomic (PGx) testing is increasingly entering mainstream clinical practice and is of great interest to patients and providers [1]. Numerous healthcare systems are implementing PGx programs [2,3,4,5,6,7,8], and statewide initiatives to implement PGx testing are also underway [9,10]. As the clinical utility and uptake of PGx has grown, conversations about PGx have shifted from “should we do PGx testing?” to “how should we best implement PGx testing?” As guideline-producing groups, such as the Clinical Pharmacogenetics Implementation Consortium [11], develop recommendations for how to best apply the scientific evidence, and as clinicians and implementation scientists [12] develop best practices for clinical implementation, there is one critical voice that must be heard: the patient. Understanding how patients perceive PGx is essential for providers to be able to anticipate their questions, concerns, and/or needs and to inform the counseling that clinicians provide prior to and after PGx testing.

The subject of patient counseling for PGx testing has received little attention. Patient and provider literacy for genetics in general has been described as insufficient and is frequently cited as a barrier to PGx implementation [13,14]. Moreover, specific PGx literacy needs have, to our knowledge, never been empirically identified outside of one study that examined the impact of objective numeracy on accurate interpretation of PGx results [15]. Given the paucity of direct research on PGx patient counseling, we conducted a scoping review of published literature on patient experiences with PGx testing.

The purpose of this review is to provide an overview of the attitudes, beliefs, and experiences with PGx testing among patients and the general public. Specific objectives were to (1) conduct a systematic search of published literature for assessments of patient knowledge of PGx, (2) perform a thematic analysis of qualitative and quantitative reports of patient experiences, and (3) discuss implications of the analysis for patient counseling for PGx testing.

## 2. Materials and Methods

Given the varied nature of patient experiences with PGx testing and the predominantly qualitative nature of the data, a scoping review was deemed to be the best methodology for our analysis, as opposed to meta-analysis [16]. Our protocol followed published guidance from the Joanna Briggs Institute on the conduct of scoping reviews [17] and adheres to the Preferred Reporting Items for Systematic Review—Extension for Scoping Reviews checklist [18]. The protocol was registered on 21 November 2021 in the Open Science Foundation Registry (https://osf.io/registries/discover; doi:10.17605/osf.io/gqfky).

### 2.1. Literature Search Strategy and Eligibility Criteria

A systematic PubMed search was performed on 28 October 2021 using the discrete search string “(patient OR consumer OR public) AND (Pharmacogen*) AND (literacy OR education OR knowledge OR understanding OR perception* OR perspective* OR view* OR attitude*)”. Filters on the search included English language, abstract included, and published after 1 January 2010. The rationale for this date is that the early 2010s marked the launch of several commercial multi-gene panels that soon became the industry standard approach and represents a transition point to more “modern” approaches to PGx testing. Articles meeting search criteria underwent title/abstract review. Full texts were downloaded and evaluated by the author (J.D.A.). Questionable articles were refereed by the senior author (J.R.B.).

Eligible articles represented studies that enrolled patients, consumers, undergraduate students, or the general public. Studies must have queried individuals about PGx testing but participants did not have to receive testing to be eligible. Eligible study designs included closed-ended surveys, open-ended surveys, semi-structured interviews, focus groups, or qualitative reviews of primary data sources.

Studies of populations that included healthcare providers, health professional students, and other stakeholders (e.g., government, laboratories, managed care, etc.) were excluded. Studies that queried individuals about disease risk genomics, somatic tumor testing, or direct-to-consumer testing were also excluded. Ineligible study designs included systematic reviews, meta-analyses, clinical outcomes trials, and clinical guidelines. Studies that reported unanchored Likert-style questions (e.g., 4.1 out of 5 on patient satisfaction) without reporting summary statistics (e.g., 80% of patients were somewhat or very satisfied) were excluded. Unpublished data, preprints, and grey literature were also excluded. Given the broad, qualitative nature of eligible articles, numeric ratings of article quality were not performed.

### 2.2. Data Elements

For each eligible article, the following characteristics were extracted: sample size, study population, study methodology, and whether study participants received PGx testing (yes/no—studies with mixed PGx tested and control populations were coded as “yes”). Data elements related to patient knowledge, attitudes, beliefs, and/or experiences with PGx testing were extracted. Due to the broad study designs included in the scoping review, data elements for a given article could include:

- Direct quotes from participants (e.g., “I was satisfied with my PGx testing”).
- Quotes from participants that are summarized or paraphrased by study authors, if no representative direct quote was present (e.g., “Many patients reported satisfaction with PGx testing”).
- Statistics from specific questionnaire items (e.g., “80% of patients were satisfied with PGx testing”).

### 2.3. Data Extraction and Thematic Analysis

Data elements were extracted manually into AtlasTi Web [19], a standard tool for qualitative review. An initial codebook was developed based on review of a subset of articles by J.D.A. This codebook was developed, reviewed, and refined by the author group. After the codebook was finalized, thematic analysis of the entire dataset was conducted independently by J.D.A. and A.L.P. Discrepancies in coding were discussed between J.D.A. and A.L.P., with J.R.B. acting as tiebreaker.

## 3. Results

### 3.1. Selection of Sources of Evidence

**Figure 1:**
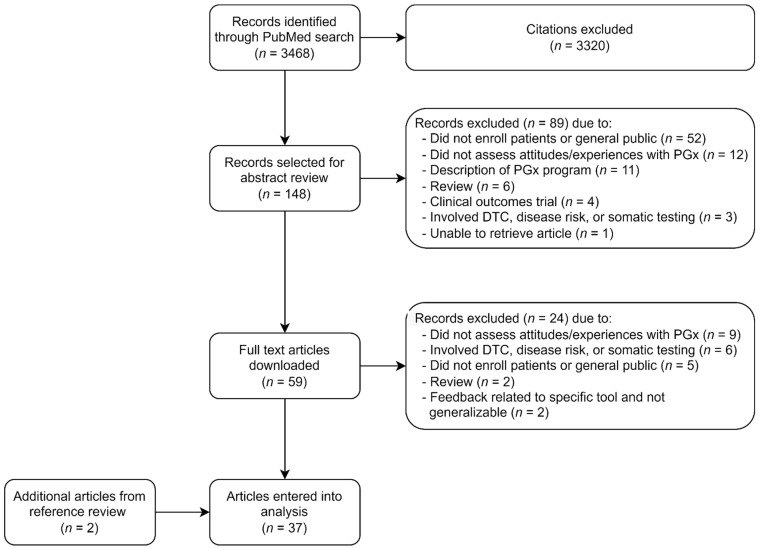
Review flowchart. Abbreviations: PGx, pharmacogenomic; DTC, direct to consumer.

The PubMed search conducted in October 2021 identified 3468 potentially relevant citations. After title screening, 148 records were selected for abstract review. Only one article could not be retrieved and was not included in the review [20]. After abstract review, 59 full text articles were downloaded. Of these, 35 met eligibility criteria and were included in the analysis. An additional two articles were identified upon reference review of eligible articles and also included in the analysis, for a final total of 37 articles [1,21,22,23,24,25,26,27,28,29,30,31,32,33,34,35,36,37,38,39,40,41,42,43,44,45,46,47,48,49,50,51,52,53,54,55,56]. Figure 1 summarizes the identification and selection process for inclusion.

### 3.2. Characteristics of Sources of Evidence

**Figure 2:**
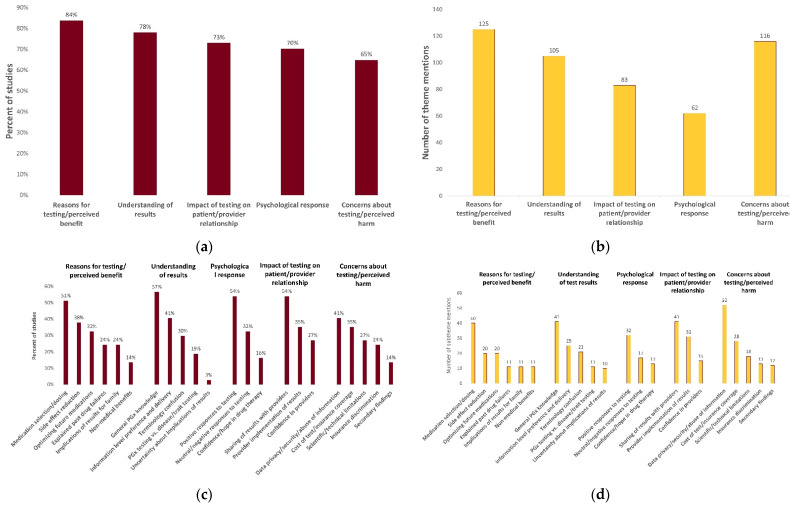
Distribution and frequency of themes and subthemes. (**a**) Percent of studies expressing each theme; (**b**) number of theme mentions across included studies; (**c**) percent of studies expressing each subtheme; (**d**) number of subtheme mentions across included studies.

Characteristics of eligible articles are reported in Table 1. All included studies were published between 2010 and 2021, with 49% (18/37) published since 2019. The total sample size across all studies was 6252 participants from a diverse range of populations. Forty-nine percent (18/37) of studies enrolled participants who had previously received PGx testing or received PGx testing as part of the study. Study methodologies included closed-ended questionnaires (*n* = 12) focus groups (*n* = 11), semi-structured interviews (*n* = 7), open-ended questionnaires (*n* = 3), or a mixed-methodological approach (*n* = 3). One retrospective qualitative review of correspondence between patients and genetic counselors was also included [52]. A total of 488 data elements were extracted from the included studies. Following thematic analysis, five major themes emerged and 22 subthemes emerged (Table 2). The frequency and distribution of these themes and subthemes is illustrated in Figure 2.

### 3.3. Theme 1: Reasons for Testing/Perceived Benefit

#### 3.3.1. Subtheme: Improved Medication Selection/Dosing

The most common theme observed across studies was an exploration of the reason for testing/perceived benefit. Participants across studies commonly identified the potential for improved medication selection/dosing as their primary motivation for testing. A large survey of the general public by Haga et al. [1] found that >90% of individuals would be likely to undergo PGx testing to predict drug effectiveness or initial dosing of a medication. This theme was also observed in participants who had received PGx testing. For example, 100% of the 150 participants in the study by Lemke et al. [33] identified helpfulness in optimizing treatment as a factor in their decision to undergo testing. Importantly, participants often spoke of testing as being able to identify the “best” medication, indicating a belief that PGx may be able to identify the one treatment that would provide the most optimal outcome for individuals. Over 75% of participants in both Haga et al. [29] and Daud et al. [30] agreed that testing would help their physician “select the best medication for them.”

#### 3.3.2. Subtheme: Side Effect Reduction

Reduction in side effects was another common expected benefit of PGx testing. Most (78%) of participants in the study by Olson et al. [35] somewhat or strongly agreed that using PGx results when prescribing medication will reduce their risk of side effects. Studies by Haga et al. [1] and Daud et al. [30] reported similar percentages of individuals interested in this benefit (73% and 72%, respectively). As one participant in Deininger et al. [39] stated, “I would definitely agree (to PGx testing). I’m the type of person that gets those weird side effects that no one else gets.”

#### 3.3.3. Subtheme: Optimization of Future Medications

In addition to optimizing current or imminently prescribed medications, in ~1/3 of the included studies, participants also spoke of the value of PGx testing for future medication decisions. Over 80% of the participants in Lemke et al. [33] agreed that PGx testing would be helpful to them in the future. For example, one participant in Truong et al. [53] stated, “I’d like (the PGx results) for every time I get prescribed a new medicine or change.”

#### 3.3.4. Subtheme: Explanation of Previous Med Failures and Non-Medical Benefits

Other notable benefits of testing did not involve immediate or future improvement in medication outcomes. Eleven studies identified that patients valued how PGx test results could contextualize previous medication experiences. Nearly 70% of the participants in Lemke et al. [33] felt “more validated about previous medication experiences” after undergoing PGx testing. Some participants described self-curiosity as a reason for testing. For example, Waldman et al. [45] stated, “Many participants expressed that the PGx testing experience had personal utility, even when (healthcare providers) did not act on medication suggestions. These participants expressed the value they placed on information and knowledge acquisition.” Others underwent PGx testing for altruistic reasons (e.g., a desire to help further research). Finally, while not necessarily the primary reason for testing, many participants expressed interest in the potential benefits of PGx for family members with health conditions. For example, one participant in Schmidlen et al. [52] queried, “I need info on my *CYP2C9* results and how they are genetically carried to my children. I have a child in critical care that may need this info.”

### 3.4. Theme 2: Understanding of PGx Concepts and Results

#### 3.4.1. Subtheme: General Pharmacogenomics Knowledge

General knowledge of genomics, pharmacology, and PGx varied widely across patient populations. While 88% of respondents in Haga et al. [29] “reported that they understood how genetic testing can be used in healthcare very well or somewhat well, and while a substantial number of participants (64%) in Pereira et al. [43] were confident of their ability to understand genetic information, participants often struggled when actually presented with test results. Less than half of participants (45%) in Olson et al. [35] were “a little” or “not at all” confident in being able to explain their PGx results to a friend or family member. Participants sometimes struggled to identify how genomic information could be applicable to specific medication classes, such as antidepressants. In contrast, 95% of participants in the study by Liko et al. [49] could describe the PGx test. Importantly, and related to reasons for developing approaches for PGx education, these participants had undergone counseling by a pharmacist before and after receiving their results.

#### 3.4.2. Subtheme: Differences in Information Level Preference and Delivery

Participants were often split on the depth and breadth of information they wanted to receive with respect to their PGx results. As Mills et al. [38] noted, “Two patient participants indicated inclusion of genotype information was not useful; however, another stated that he ‘wants to know everything (because the more information) you give, the more knowledge patients have…” In other cases, as Jones et al. noted [36], “Some patients requested to know everything about the result, ‘I’d like to know as much as I can;’ however, after reviewing the mock report, they thought that some sections of the report had too much information.”

This difference in information level preference may relate to patients’ preferred method of information delivery. Some participants stated that they preferred to receive PGx information from a healthcare provider, while others preferred to look it up themselves. Lemke et al. [33] found that 40% of participants looked up additional information about their results, while 35.7% wanted additional follow-up from a provider to discuss their results. Nearly 1 in 8 participants in Lemke et al. made changes to their medication regimen on their own.

#### 3.4.3. Subtheme: Terminology Confusion

The terminology commonly used in the PGx field and on test reports often posed a barrier to patient understanding. As an example, only 17% of participants in Daud et al. [30] were aware of the meaning of the term “pharmacogenetics.” While some participants were able to break apart the word, others found it incomprehensible (“I don’t even know what that means.” [49]). Patients in the 2018 paper by Mills et al. [38] reviewing PGx educational material, “expressed concern about potentially confusing terminology. Two patient participants felt the term ‘metabolize’ needed a better definition, with one pointing out the risk of confusing it with ‘metabolism of food.’ One also worried that some patients may not be familiar with the term ‘enzyme.’” All participants in Asiedu et al. [46] “expressed concerns about the medical/technical terms such as ‘metabolize,’ ‘metabolic status,’ and ‘phenotype’.

#### 3.4.4. Subtheme: Confusion between PGx Testing vs. Disease/Trait Testing

Another common (and persistent) source of confusion was the difference between PGx testing and disease risk or trait testing. Trinidad et al. [28] noted this refrain throughout the seven focus groups they performed: “most understood the purpose of genetic testing to be predicting one’s susceptibility to heritable illness.” Lee et al. [32], talking to participants who had not received PGx testing, noted that the group “universally confused PGx with disease risk testing, in some cases continuing even after hearing a definition of PGx and its applications.” Likewise, Dressler et al. [40] noted, “Despite the informed consent process specifically indicating that testing would not provide any disease risk information, 60% of participants on the pretest survey expected to receive results on cancer risk. Although this misconception significantly decreased in the post-testing survey, we still observed this expectation in 49% of respondents.”

#### 3.4.5. Subtheme: Uncertainty about Implications of Results for Care

One particular area of confusion that deserves special mention is patient uncertainty about the implications of their results for care. While this concept was only identified in the Schmidlen et al. [52] paper, it nevertheless came out strongly. Schmidlen et al. retrospectively reviewed PGx-related genetic counseling requests as part of a large trial and found that 54% of participants requesting genetic counseling for a PGx results were seeking general assistance with understanding their results. Commonly, participants alluded to uncertainty regarding whether they should or should not be taking a given medication based on their study result or had questions about how their results applied to other medications.

### 3.5. Theme 3: Impact of PGx Testing on the Patient/Provider Relationship

#### 3.5.1. Subtheme: Sharing of Results with Providers

Most participants desired to have their PGx results shared with their healthcare provider. In Haga et al. [29], 92% of participants said that they would share the results with other prescribers. In Lanting et al. [48], 74% of respondents planned to discuss results with their physicians, 37% reported doing so at follow up in a regular appointment, and 5% did so at a separate appointment. Many participants discussed the question of who would be the best provider to prescribe, interpret, and implement the PGx results. Most participants agreed that their primary care physician should have access, and many also wanted specialty physicians to have access if it was relevant to their practice. Moreover, participants often had an expectation that PGx results would immediately become a part of their medical record and therefore both accessible and actionable to all physicians.

Participants were more hesitant to share results with their pharmacist. While some participants felt their pharmacist was in the best position to monitor and act upon this information, many were unclear about whether pharmacists were adequately trained or appropriately positioned to act upon the information. Only 6.3% of participants in Waldman et al. [43] reported that they shared and discussed their PGx results with a pharmacist. Waldman et al. [45] stated, “of those who did not disclose their PGx results to a pharmacist (93.7%, *n* = 30), one participant stated that they “would probably rely on his or her doctor to prescribe the right medications based on the results,” and another stated that they “hadn’t thought of it prior to this survey”.

#### 3.5.2. Subtheme: Provider Implementation of Results

When PGx results were shared with healthcare providers, the response from providers varied. In Lanting et al. [48], 71% of conversations with healthcare providers were scored by participants as “very good,” while 13% were scored as “very bad.” In Waldman et al. [45], 87.1% of participants reported no changes to their medication regimen based on their PGx results. Of these, 44.4% reported that their medication regimen was already consistent with their PGx results, while 18.5% reported that their healthcare provider did not feel the need to change their medical care because of their PGx results. Many participants reported that providers were skeptical of the results or did not know how to properly implement them.

On the other hand, an additional concern raised by some participants was the potential for providers to rely on PGx testing to the exclusion of other factors. Some identified that genetic variation was only one factor in medication response and wanted their providers to practice a more holistic approach to medication selection.

#### 3.5.3. Subtheme: Confidence in Providers

Participants generally reported trusting their healthcare providers about the decision to undergo PGx testing. A total of 59% of participants in both Haga et al. [1] and Daud et al. [30] underwent testing based on physician recommendation. In Pereira et al. [43], 75% of participants felt comfortable if their physician recommended genetic testing to guide their healthcare. Participants also expressed more confidence in providers who did take the time to understand and implement their PGx results. In Lemke et al. [33], 57% of participants agreed that they would be more likely to take medications prescribed by the provider if those decisions were informed by PGx testing. Participants felt that PGx testing made providers more informed and more “cutting edge”.

### 3.6. Theme 4: Psychological Response to PGx Testing

#### 3.6.1. Subtheme: Positive Psychological Responses to Testing

Participants first learning about PGx testing commonly had a positive response. In a survey by Haga et al. [1], 65% of respondents were extremely or somewhat likely to undergo PGx testing after being informed of the risks. This number increased to 82% after learning more about the uses of PGx testing. This positive response frequently remained after receiving their PGx results. Participants in Lanting et al. [48] reported that knowing their PGx profile was “comforting” (89%), “useful” (92%) and that PGx testing “did not frighten them” (88%). In Olson et al. [35], 60% of respondents said they would encourage others to get *CYP2D6* testing done, while 65% somewhat or strongly agreed that if more PGx tests became available they would ask their healthcare provider to order them.

#### 3.6.2. Subtheme: Confidence/Hope in Medication Therapy

One frequently repeated positive response was that of improved confidence and/or hope in medication response as a result of PGx testing. Lemke et al. [33], querying individuals who underwent PGx testing, found that 73% of them felt more confident that the medications prescribed will not cause side effects. Some individuals brought up a tendency for healthcare provider to discount or pathologize patients’ experiences of medication side effects and expressed hope that PGx testing would provide a physiological basis for those experiences. As one participant in Frigon et al. [41] related, “…if an individual was not responding to a treatment, then it meant that the individual was having somatic symptoms. That was the term we were using. I am happy that you are bringing this up because this might avoid people being told that they are having somatic symptoms.”

#### 3.6.3. Subtheme: Neutral/Negative Responses

Positive responses to PGx testing were not universal. Some participants expressed negative responses about undergoing testing, including feelings of skepticism that results would be helpful or unspecified fears. Some individuals who received PGx testing felt underwhelmed about the results. One-third of individuals in Haga et al. [29] indicated they felt nervous or anxious about their results. Others felt more neutral about the subject and either did not remember their results or did not feel PGx testing strongly impacted their life either positively or negatively. Interestingly, one participant specifically mentioned a concern relating to informed consent: “I don’t feel comfortable with my grandmother coming in here and being informed about what she’s signing up for.” [25].

### 3.7. Theme 5: Concerns about PGx Testing and Perceived Harm

#### 3.7.1. Subtheme: Data Privacy/Security/Abuse of Information

The primary concern shared by participants across 41% of the included studies was that of data privacy and security. Indeed, 40% of participants in Lemke et al. [33] indicated concern about privacy of data, while nearly 80% of respondents in the survey by Haga et al. [1] were “not very” or “not at all” likely to have PGx testing if there was a chance their DNA sample or test result could be shared without their permission. Participants expressed concern that information would be misused or inappropriately accessed, and could lead to targeting by pharmaceutical companies or misuse by employers, law enforcement agencies, or the government. Even within the context of the healthcare system, there were concerns about data privacy. However, other participants expressed a lack of concern about PGx information, and expressed willingness for health care providers to have access to the information as long as it was relevant to their practice.

#### 3.7.2. Subtheme: Insurance Discrimination

A specific concern related to abuse of information was insurance discrimination. Over 30% of the participants in Lemke et al. [33] indicated concern about the potential for discrimination, while about three-quarters of respondents in O’Daniel et al. [23] “indicated fear of discrimination by employers and health insurers” as a major reason to not undergo PGx testing.

#### 3.7.3. Subtheme: Cost/Insurance Coverage

Another major concern of many participants was the out-of-pocket cost and/or insurance coverage of the test. Lack of insurance coverage for testing was a “very important” or “somewhat important” reason for declining PGx testing among respondents in O’Daniel et al. [23]. In contrast, if the entire cost of testing was covered by insurance, 89% of respondents in Gibson et al. [31] said that they would be very likely to get one. Many participants speculated on the cost/benefit ratio of the testing, while others expressed concern over issues of equity if the testing was too expensive or led to medication recommendations that were unaffordable for a portion of the population. Others questioned the societal benefit of large-scale PGx testing.

#### 3.7.4. Subtheme: Scientific/Technical Limitations

Some participants were worried about the scientific and technical limitations of the testing. These limitations included topics such as testing accuracy, convenience of testing (e.g., concerns about blood draws), and the breadth of medications included. One participant in Lee et al. [32] asked, “How accurate is this genetic testing related to medications? Is there enough track record? Is it on target?” Post-testing, some participants expressed concern about results either being unhelpful or not aligning with their prior experiences with medications. For example, a participant in Liko et al. [49] commented, “They said green about a drug that I had stopped years past that didn’t work. So I think that was the thing, like one of the greens was like no, that’s not a green.”

#### 3.7.5. Subtheme: Secondary Findings

Some participants worried about secondary findings as a result of undergoing PGx testing. This was often connected to the concerns about insurance discrimination, i.e., that PGx testing would turn up additional findings that would then result in insurance discrimination. In Madadi et al., [22] there were “several reservations, spontaneously expressed by 15% of participants, toward some perceived facets of genetic application, namely, mutagenesis, pregnancy termination, genetic engineering, and/or testing for incurable genetic diseases.”

## 4. Discussion

In this scoping review, we analyzed the results from 37 articles that employed survey-style research as well as focus groups and semi-structured interviews. We identified five themes and 22 subthemes (Table 2) that provide a comprehensive understanding of the patient perspective regarding PGx testing and its implementation in patient care.

Each of the themes identified has implications for patient care and counseling with respect to PGx testing. Regarding the theme of reasons for testing/perceived benefit, the majority of participants were interested in testing to help address efficacy and safety of medications. Many held a belief that we may call “the myth of the perfect medication,” in which PGx testing will identify the exact medication that will provide the most optimal treatment for their condition. In a recent survey conducted by the authors at a state fair, 89% of members of the general public believed that “pharmacogenomics will tell you the best medication to treat your condition,” while 67% of members of the general public believed that “when deciding what medication is best for you, your genetic makeup is more important than age, weight, or other medications you are taking” (unpublished data). This tendency toward “genetic exceptionalism” (i.e., the idea that genetic information holds special significance over and above other types of clinical data), may lead to feelings of disappointment, anger, and failure if results do not align with patient expectations (as expressed in our “psychological responses” theme). Intriguingly, some participants underwent PGx testing for reasons unrelated to medication assessment. Some were interested in the results as a means of learning more about themselves (“self-curiosity”) or for altruistic reasons, such as to provide information for family members or to advance research. PGx providers should be aware of a patient’s reasons for undergoing PGx testing and tailor their messaging appropriately.

Regarding the theme of concerns about testing/perceived harm, we observed a general trend for participants to exhibit great enthusiasm for PGx testing initially, which tempered when they became more informed about the limitations of testing. Concerns about insurance discrimination, data privacy, abuse of information, and cost of testing were common. Here too, genetic exceptionalism may be at play, as it is unlikely that participants would be as concerned about others having access to other laboratory results (e.g., serum creatinine). While all genetic information comes with ethical, legal, and social concerns, PGx testing tends to pose fewer concerns than disease risk genomic testing [57]. Nevertheless, providers should ensure that patients are thoroughly educated on the entire chain of custody of their genomic information, how their results will be secured, and who will have access to the information. Test-specific limitations and talking points (e.g., allele coverage, secondary findings, etc.) should also be thoroughly addressed prior to the patient undergoing testing.

Regarding the theme of the impact of testing on the patient/provider relationship, participants had variable experiences with PGx testing in the context of the patient/provider relationship. Patients thought highly of providers who offered PGx, feeling that it would improve their ability to select medications that would be safe and effective. Use of PGx also signaled that providers were interested in practicing cutting edge medicine. Patients often had an expectation that providers would know how to interpret and implement their PGx results, and that results would be shared among their different healthcare providers. They frequently expressed disappointment when this was not the case. These findings speak to the need for comprehensive PGx education for all providers and for systems that allow PGx results to be shared among all providers involved in a patient’s care.

Patients also differed on which providers they felt would be the best for implementing PGx testing. Some preferred that it come from their primary care provider, while others wanted disease state specialists or genetics specialists to deliver the information. While pharmacy is often involved in driving PGx implementation within health systems [58], most participants did not feel that pharmacists would be the most appropriate individuals to implement PGx, possibly due to unfamiliarity with the role and education of a clinical pharmacist. This is in contrast to evolving data suggesting that pharmacists may be optimally positioned to educate patients and provide therapeutic recommendations that incorporate PGx data [59,60,61]. This speaks to the need for greater advocacy to improve the public’s perception of the roles of the pharmacist beyond traditional dispensing activities.

Patient understanding of PGx testing remains low. Patients struggled with nomenclature and frequently conflated PGx testing with disease risk genetic testing. These findings underscore the importance of pre-test education and counseling so that patients are able to make informed decisions about whether or not to undergo testing, how to interpret results, and to have an informed discussion with their provider about PGx-informed medication decisions. One open question for the field is how to assess patient PGx literacy to ensure adequate informed consent. Genomic literacy assessments exist in the disease risk literature [62,63,64,65], but none of these assessments include PGx-related content. The development of a PGx-specific knowledge assessment would greatly assist clinicians and researchers to assess the efficacy of PGx educational interventions.

One intriguing finding of our study was the subtheme of information level preference and delivery. There was wide variability between participants in terms of the depth of information they requested about PGx. Moreover, we found that for some participants, their information level preference was dynamic and changed based on the information presented and its salience to their questions. This poses a challenge for developers of PGx test reports, which typically require standardized, relatively static report formats. Future work should examine novel test formats that allow for scalable or customizable information levels tailored to patient (and clinician) preference.

Our paper adds valuable context to the evolving knowledge of what is important for PGx patient counseling. Many of the knowledge gaps, misunderstandings, and concerns that participants identified could be mitigated by robust pre- and post-test counseling. In Table 3 and Table 4, we outline several pre- and post-test counseling points that should be considered based on the themes we identified in our analysis. In a recent article that was published after we completed our analyses, Wake and colleagues [66] compared pre-test counseling themes across four pharmacist-led PGx clinical practices. The authors identified several similar themes across the four sites, including benefits, limitations, and concerns/risks of PGx testing. The article also outlines core elements of a PGx counseling session using the acronym PGX-DRUGS (Purpose and benefit of PGx testing, Genetic concepts, X-amples (“examples”), Drawbacks of PGx testing, Risks and concerns, Understand patient’s view of PGx testing, Game plan and process, and Sharing PGx results). The themes identified from this “expert opinion” perspective largely correspond to those identified in our “patient opinion” research and provide independent validation of our findings.

### Strengths and Limitations

To our knowledge, this is the first scoping review to summarize and synthesize patient attitudes and experiences with PGx testing among patients and the general public. We used a broad search strategy, encompassing nearly 3500 articles, thus ensuring high sensitivity. Our analysis used multiple independent reviewers to ensure reliability of findings.

A major limitation of the analysis includes the reliance on reports of qualitative findings, rather than direct analysis of the qualitative transcripts themselves. Reporting on qualitative results necessitates interpretation, selection, and simplification of content—all possible avenues for author bias to affect results. Further, some quotations were presented without their accompanying context, making their classification ambiguous at times.

## 5. Conclusions and Future Directions

Pharmacogenomics represents a first step towards mainstream genomic medicine; thus, assessing and improving patients’ PGx literacy is a critical factor that must be addressed. Our analysis has relevance for development and standardization of patient counseling for PGx, but more research is needed on patients’ PGx educational needs. An open-source patient-focused education curriculum would allow for more efficient development and delivery of PGx counseling. This curriculum needs to be standardized yet scalable to individual information level preference. Additionally, there is a need for validated knowledge assessments that can be used to gauge the effectiveness of PGx patient counseling. Together, these steps will allow us to provide patients with the right PGx education, at the right level, at the right time.

## Figures and Tables

**Table 1:** Included studies and their characteristics.

Year Published	Author	Sample Size	Study Population	Study Methodology	PGx-Tested Patients Included?	PMID
2010	Haddy et al. [21]	35	General public	Focus group	No	20813335
2010	Madadi et al. [22]	62	Canadian breastfeeding mothers taking codeine	Semi-structured interview	Yes	20739920
2010	O’Daniel et al. [23]	75	Patients in a family medicine center	Closed-ended survey	No	19407441
2011	Haga et al. [24]	45	General public	Focus group	No	22047505
2012	Haga et al. [1]	1139	National phone survey	Semi-structured interview	No	21321582
2013	Shaw et al. [25]	32	Alaska natives	Focus group	No	24134351
2014	Brewer et al. [26]	320	Women with breast cancer (85% taking tamoxifen)	Open-ended survey	No	24457521
2014	Chan et al. [27]	222 warfarin patients; 224 general public	Singaporean warfarin patients/general public	Closed-ended survey	No	24468050
2015	Trinidad et al. [28]	61	27 patients taking antidepressants, 17 patients taking carbamazepine, 17 healthy patients	Focus group	No	26057686
2016	Haga et al. [29]	17	PGx-tested primary care patients	Closed-ended survey	Yes	27648637
2017	Daud et al. [30]	219	Formerly pregnant women in Netherlands	Closed-ended survey	No	28410576
2017	Gibson et al. [31]	27	Customers of an independent community pharmacy	Closed-ended survey	No	28112585
2017	Lee et al. [32]	9 PGx tested; 13 traditional care	PGx-tested primary care patients vs. traditional primary care patients	Focus group	Yes	28267054
2017	Lemke et al. [33]	57	Unspecified PGx testing recipients	Focus group + closed ended survey	Yes	29469671
2017	Mills et al. [34]	7	General public	Focus group	No	28587070
2017	Olson et al. [35]	869	Biobank patients from RIGHT protocol receiving *CYP2D6* results	Open-ended survey	Yes	28055020
2018	Jones et al. [36]	13	Geisinger biobank participants	Semi-structured interview	No	29316365
2018	Lee et al. [37]	703	Korean adults who visited community pharmacies or public healthcare centers	Closed-ended survey	No	29451916
2018	Mills et al. [38]	4	General public	Focus group	No	30214267
2019	Deininger et al. [39]	36	Solid organ transplant patients	Focus group + semi-structured interview	No	31755847
2019	Dressler et al. [40]	51 (pre-test) 49 (post-test)	Unspecified PGx testing recipients	Closed-ended survey	Yes	30983513
2019	Frigon et al. [41]	30	Patients in pharmacies in Quebec	Focus group	No	31190623
2019	Haga et al. [42]	99 (full completion), 16 (partial completion)	Patients subscribed to a PGx laboratory’s email list	Closed-ended survey	Yes	31190624
2019	Pereira et al. [43]	1327 (baseline); 860 (follow-up)	Patients from US, Canada, and Korea enrolled in TAILOR-PCI study	Closed-ended survey	Yes	30724853
2019	Truong et al. [44]	10 PGx-tested; 10 traditional care	Patients from 1200 Patients Project	Closed-ended survey	Yes	31490020
2019	Waldman et al. [45]	37	Unspecified PGx testing recipients	Open-ended survey	Yes	30983503
2020	Asiedu et al. [46]	24	Participants in the RIGHT protocol	Focus group + semi-structured interview	Yes	32292118
2020	Johnson et al. [47]	10	Patients at a federally qualified health care center	Closed-ended survey	Yes	32269458
2020	Lanting et al. [48]	165	Dutch outpatients	Closed-ended survey	Yes	33371313
2020	Liko et al. [49]	20	Psychiatric outpatients	Semi-structured interview	Yes	32583391
2020	Png et al. [50]	14	Hospital admission for an ACS and underwent PCI, on ticagrelor	Semi-structured interview	No	32524842
2020	Rigter et al. [51]	21	Dutch primary care patients	Focus group	No	32076434
2020	Schmidlen et al. [52]	80	Coriell Personalized Medicine Collaborative participants who requested genetic counseling	Retrospective qualitative review	Yes	32340147
2020	Truong et al. [53]	10	Participants in the 1200 Patients Project	Focus group	Yes	33017129
2021	Bright et al. [54]	19	Patients from community pharmacies taking clopidogrel or an SSRI	Semi-structured interview	No	32741696
2021	Meagher et al. [55]	54	Participants in the Mayo Clinic Biobank	Focus group	Yes	32919825
2021	Stancil et al. [56]	17	Adolescents who received PGx testing as part of clinical care	Semi-structured interview	Yes	33849282

Abbreviations: PGx, pharmacogenomic; ACS, acute coronary syndrome; PCI, percutaneous coronary intervention; SSRI, selective serotonin reuptake inhibitor.

**Table 2:** Themes, subthemes, and representative quotations from included studies.

Theme	Subtheme	Representative Quotations
Reasons for testing/perceived benefit	Medication selection/dosing	“You could jump off anywhere downtown and get to a store, but you want to get off closer to the store you’re going to.” [28]. “It helped (the provider) decide on a new—I think it was an SNRI that we chose…And that actually was helpful for quite some time. For a good, I would say, good 18 months, it was probably kind of pretty useful. So that was good.” [49]. ‘‘I would (have) the testing done to determine the best medication—the medication that is best for you based on your genetic makeup.’’ [24].
	Side effect reduction	“I would definitely agree (to PGx testing). I’m the type of person that gets those weird side effects that no one else gets.” [39]. “After my testing and results, my medications were changed and I did notice that I no longer had my ankles swelling. Even my family doctor thought prior to testing it was something else and had me on water pills for a short time to reduce the swelling. It was not until the testing was done and the medications were changed that I noticed results.” [45].
	Optimization of future medications	“I’d like (the PGx results) for every time I get prescribed a new medicine or change.” [53]. “…very useful information that I will be able to apply to my own personal health management decisions for the rest of my life.” [45].
	Explained past drug failures	“I have had side effects, uncomfortable side effects. And then when we did the test, we found out why I probably had those reactions.” [49].
	Implications of results for family	“Many participants wanted to understand the inheritance pattern of 5-FU toxicity. This was often expressed as a question about the chance that a given family member might have the variant of interest.” [55]. “I need info on my *CYP2C9* results and how they are genetically carried to my children. I have a child in critical care that may need this info.” [52].
	Non-medical benefits	“Many participants expressed that the PGx testing experience had personal utility, even when HCPs did not act on medication suggestions. These participants expressed the value they placed on information and knowledge acquisition.” [45]. “If we don’t come in and help out, how are you guys gonna, you know, further research, if we don’t participate?” [53].
Patient understanding of results	General PGx knowledge	“What do they find out when they say ‘by my blood test’ and the picture of me? So what in my blood tells him that this drug is better that drug?” [32]. “So genetic coding, you want a piece of my blood to understand how…I work mentally. That just doesn’t add up to me.” [25]. “So basically they take your genes and look at them and compare it to different medications and look for different genomes, genes, patterns, or whatever to see which medications they think will work best for you and which medications they think won’t work for you.” [49].
	Information level preference and delivery	“For me, that works but maybe some people might want a little bit more explanation if they don’t have the same comfort level with pharmacists and doctors and the same trust level.” [54]. “I really don’t have any problems with it. I don’t understand something, I look it up. It’s so simple.” [53]. “I’d rather just get told; someone just to tell me (face-to-face) because I don’t know…It would be easier for me to comprehend.” [39].
	Terminology confusion	“Well it’s obviously pharmaceutical, and ‘genomics’ is kind of an open-end—I know it has to do with DNA. But as far as anything deeper, I don’t know.” [39]. “I’m stumbling on some of these that terminology, that genetic thing, genetic makeup. What is that? I’m a man, I don’t do makeup.” [46]. “Name all genes. Not *CYP2D6*—call it CARL. That, I can remember,” [35]. “It is unclear what AA, GG, TT, etc. mean.” [52].
	PGx testing vs. disease/trait testing	“It [helped] in my situation realize that I don’t have many health issues.” [45]. “I came in with the idea that this is a testing of your genes, your genetic makeup, to find out if you are more predestined for a certain disease…. Life-threatening things, that’s what I thought it was all about.” [28].
	Uncertainty about implications of results	“I have high cholesterol and have had adverse effects with other statins. I have not used simvastatin and would like to discuss if my results recommend trying this drug.” [52]. “I have a family history of stroke (mother) and she is currently on this medication. In the future, if this drug is offered, should I decline as it does not look effective in my case?” [52]. “I was wondering if the study will be doing tests for which antibiotics might not work with my genes?” [52].
Psychological responses to results	Positive responses to testing	“I think it’s a great idea. Who wouldn’t want more information about the proper medication to take?” [28]. “I cannot say enough good things about this being made available to us as patients and in healthcare. The opportunities are so profound, when this knowledge is applied appropriately.” [45]. “His PGx results helped his ‘peace of mind’ even though he was ‘disappointed’ that the results did not provide an answer about his medication of interest (ondansetron).” [56].
	Neutral/negative responses to testing	“…if I was going to do this genetic testing it would have to be over more grave circumstances… If I don’t do it, I’m going to die.” [32]. “I wasn’t so sure anything was going to help. But I figured it couldn’t hurt I guess. Like, okay it’s not painful. Go for it. But I don’t think I had high hopes particularly.” [49]. “Because there wasn’t clear evidence to me, pointing to selection of drug…I don’t think… that’s what made the critical difference. I think it was my therapist that made the critical difference.” [49].
	Confidence/hope in drug therapy	“…if an individual was not responding to a treatment, then it meant that the individual was having somatic symptoms. That was the term we were using. I am happy that you are bringing this up because this might avoid people being told that they are having somatic symptoms…” [41]. “(I liked knowing) that I’m not as problematic (of a) medicine taker as I thought I was.” [56]. “I was really scared about starting a medication and then having to change it so we figured out what would work best for me and (it) made me more relaxed and trusting the process more…” [56].
Effect on patient/provider relationship	Sharing of results with providers	“I guess it gets associated in my medical record… I think this information ought to be in there.” [36]. “Definitely my transplant nephrologist (should have access). I feel that team should know since they’re the ones that are prescribing me all these medications…I’d be okay with majority of my physicians having that information if it would, in some way, benefit them in treating me.” [39]. “Well, my most frequent interaction is with my pharmacist. So if this is about the medications and how my body handles them, the pharmacist.” [39]. “I don’t know much about pharmacy, about the pharmacist… I don’t know if he would know what drugs do what to certain people with certain types of genetics because I don’t know his training or expertise. So maybe I might go with the genetic, possibly lean towards the genetic counselor.” [54]. “But I think a pharmacist in itself, is too commercial to do such things (order a PGx test and adjust treatment accordingly). A blood drawing station or so (could do that), okay, or the GP himself, but a pharmacist absolutely not.” [51].
	Provider implementation of results	“When I did share it with my doctor, she said, ‘Well, we can put this on file, but we won’t have any way to reference this.’ She didn’t seem concerned at all. She didn’t seem alarmed. And to me, that killed the momentum of, ‘Okay, I need to share this,’ or anything along that nature.” [55]. “My doctor did not take the-the copy that I put down there for him. He did not take it. But he did look at it. You know, he read it and everything—and the nurse did, too. But, uh—I don’t think he put it in my history, so it’s gonna be up to me to, to mention it.” [55]. “Sounds terrible. What bothers me is that I am waiting for my specialists to treat ME, NOT my test results, treat ME, not the TEST results.” [21]. “I would be sure that both care provider and patients know that just because you are a slow metabolizer or poor metabolizer doesn’t mean that medication is off the table.” [56]. “I want to say that again, what really bothers me the most: you have something static, which is your genome, and the way medication reacts is all different. And all other kinds of physical situations that may affect that medication. And because (the genome is) static, would the doctor be more inclined to say … ‘I’m sorry, that’s what the test says’? [28].
	Confidence in providers	“They also like to educate you. I come here because I feel like they educate me and they go through all my options, and then they make a decision based on what’s going to be appropriate.” [53]. “I see it as positive…If I have a doctor who’s using this information… they’re staying on the front end of available information and advances.” [32]. “It would make the pharmacist more informed and again another double, triple check before they’re handing me the medications.” [32].
Concerns about testing/perceived harm	Data privacy/security/abuse of information	“I think people might lose a little bit of trust there if they feel like someone’s making a profit off their information. If they’re making a profit off making drugs that help them, I think that would be different. Let’s see. Would I personally have a problem with that? Not necessarily.” [24]. “I have never agreed with total government control of knowledge, even if it will help me and my family.” [21]. “We’re giving away extensive information and we don’t know what it means. Right now I do this in an environment of a sense of trust.” [32]. “The privacy part shouldn’t matter. If that saves your life…someone else being nosy can save my life, I would appreciate that.” [32]. “I feel like the pharmacists all know what type of medication you’re on, so it doesn’t really add another worry about privacy to just do that extra test.” [54].
	Cost of test/insurance coverage	“How significant is the difference between the different drugs…is it enough to warrant all the extra testing cost or price difference in drugs?” [32]. “We have a financial concern but, above all, we want to be healthy.” [41]. “We would not accept a medicine for the rich and one for the poor for the DNA test.” [41]. “If you’ve like been through a bunch of drugs and you haven’t found something that works it would probably be beneficial… If you’re like trying to weigh the cost of spending 6 more months going through a bunch of drugs…if you’re like trying your first drug, like I don’t think it would be like worth it to spend right away… unless it’s like totally covered by insurance.” [49]. “It sounds like testing that could help a few people when maybe that money could be spent to help a lot more people.” [25].
	Scientific/technical limitations	“How accurate is this genetic testing related to medications? Is there enough track record? Is it on target?” [32]. “Satisfactory but disappointed that the medications I take are not listed in the results.” [45]. “But if it were costly, if it were painful, if it were difficult to do—like we are saying, if I had to take another step—instead of just going to the pharmacy, I had to take a test, it might just make me delay it if it takes a hassle.” [54]. “They said green about a drug that I had stopped years past that didn’t work. So I think that was the thing, like one of the greens was like no, that’s not a green.” [49]. “We were like okay, this drug that seems to be working is red so we didn’t say we’re going to stop it, you know? I think that we recognized that this was a very fallible test…. I think the ones that were red, like I liked that drug, I feel like this drug is working for me.” [49].
	Insurance discrimination	“The only thing I don’t like about (pharmacogenetic testing is), because of certain percentages, you might not be good on a certain drug, maybe, and they make this whole list of all these good drugs you can’t have. So they would refuse certain medicines to you, your whole life.” [28]. “And the insurance companies, they get a hold of your information…it’ll make premiums and everything go up.” [53].
	Secondary findings	“What if the information is something dreadful that they can’t do a damned thing about? I wouldn’t want to know that, and I would certainly want to know in advance if that was a possible piece of information, especially given the insurance considerations.’’ [24]. “I kind of want to know how much information they can get from that blood sample…If I’m using it for something very, very specific, that sort of works, but they are also getting information about my IQ, my willingness to work Monday through Friday, or my need to call in for a vacation day every three weeks, or three days? I don’t want that in there.” [28].

Abbreviations: SNRI, serotonin-norepinephrine reuptake inhibitor; PGx, pharmacogenomic; 5-FU, 5-flouoruracil; HCPs, healthcare providers; GP, general practitioner; IQ, intelligence quotient.

**Table 3:** Pre-test counseling points.

Theme	Counseling Points
Reasons for testing/perceived benefit	Understand patient’s goals of testing (efficacy, side effects, explanation of previous failures, non-medical, etc.) and tailor messaging appropriately. Temper expectations that PGx will find the “best” medication.
Understanding of results	Discuss basics of genomics and pharmacogenomics. Use accessible terminology and provide patients with documentation to help explain unavoidable jargon (e.g., drug metabolism). Clearly delineate the difference between disease-risk genomics and PGx.
Psychological response	Discuss the concept of genetic exceptionalism, which can cause patient and clinician to value PGx results more than other common laboratory tests. Prepare patients for possible psychological reactions: excitement, disappointment with normal results, discouragement if results don’t align with their experience. Prepare patients for family implications. Results may or may not have relevance for family members—parents, siblings, children.
Impact of testing on patient/provider relationship	Discuss plans for follow up, implementation, and sharing of results within the healthcare system.
Concerns about testing/perceived harm	Counsel patients on limitations of testing, emphasizing that genetics is one of many factors that can that influence drug response. Inform patients about who will have access to their results, under what conditions the information would be shared with third parties, steps taken to secure the information, and legal protections against insurance discrimination (i.e., GINA). Educate on the narrow focus of PGx to detect medication-related genetic variation, but also thoroughly discuss any possible secondary findings that may arise from the testing (e.g., Factor II/V, *UGT1A1*). Discuss methodological limitations of the test platform and the potential for undetected variants that could affect medication response. Discuss scientific limitations of our understanding of PGx—both with respect to medications covered and the current state of knowledge regarding the genetic underpinnings of medication response. Have a frank discussion with patient about the likely cost of the testing (including the “worst case scenario” if billing is unclear). Make sure the patient is comfortable with the cost of the test before proceeding.

Abbreviations: PGx, pharmacogenomic; GINA, Genetic Information Nondiscrimination Act.

**Table 4:** Post-test counseling suggestions.

Theme	Counseling Points
Reasons for testing/perceived benefit	Provide clear guidance on the implications of the results for the patient and a mechanism for them to ask additional questions. Reiterate messaging that PGx may not help find the “best” medication and place results in the proper context of multiple medication factors. Explain implications of results for future medications.
Understanding of results	Be ready to review themes discussed in pre-test counseling. Provide informational resources at multiple levels of understanding to accommodate multiple information level preferences. Provide individuals with resources for self-research and opportunities to speak with a healthcare professional.
Psychological response	Anticipate patient’s psychological response to test results (as discussed in pre-test counseling) and counsel accordingly. Discuss patient’s plans to share results with family members and outline which results may be more or less beneficial to share.
Impact of testing on patient/provider relationship	Ensure that results are delivered by someone who is well-versed in PGx and is in a position to take responsibility for result implementation: either directly by making med changes or indirectly by ensuring communication of results to other prescribers and education of those prescribers on implications of the results. Inform patient about how results will be used in their care both now and in the future, including (if available) any EHR-based informatics tools that will automatically generate medication prescribing alerts. Provide patient with a copy of results and suggestions for which providers might most benefit from access.
Concerns about testing/perceived harm	Reiterate protections for PGx information and who will have access to the information. Explain any relevant limitations of testing. Discuss any secondary findings that arise and outline a plan for next steps, if any (e.g., speak to a genetic counselor).

Abbreviations: PGx, pharmacogenomic.

## Data Availability

No new data were created or analyzed in this study. Data sharing is not applicable to this article.
